# A Randomized Intercept Survey Trial to Test the Effectiveness of Multiple Traffic Light Labels on Online Grocery Shopping Behaviors in Bahrain

**DOI:** 10.3390/nu18101645

**Published:** 2026-05-21

**Authors:** Soye Shin, Ali Shubbar Jawad, Buthaina Yusuf Ajlan, Fatema Ahmed Mohammed Isa, Amna Ghassan Alawadhi, Reem Alsukait, Eric A. Finkelstein

**Affiliations:** 1 Health Services Research and Population Health, Duke-NUS Medical School, Singapore 169857, Singapore; 2 Saw Swee Hock School of Public Health, National University of Singapore, Singapore 117549, Singapore; 3 Ministry of Industry and Commerce, Manama P.O. Box 60667, Bahrain; 4 Ministry of Health, Manama P.O. Box 60667, Bahrain; 5 Community Health Sciences, College of Applied Medical Sciences, King Saud University, Riyadh 12372, Saudi Arabia; 6 Health, Nutrition and Population Global Practice, World Bank Group, Washington, DC 20433, USA

**Keywords:** front-of-pack labeling, Multiple Traffic Light, nutrition labeling, online grocery shopping, randomized controlled trial, Bahrain

## Abstract

**Background/Objectives:** Multiple Traffic Light (MTL) front-of-pack (FOP) labels are being considered in Bahrain. We tested whether an adapted MTL label improves the nutritional quality of grocery purchases. **Methods:** In a two-arm randomized controlled intercept trial (January–May 2025), adults (≥21 years) responsible for household grocery shopping were recruited in high-footfall public venues and asked to complete a one-time shop on a tablet-based, purpose-built online grocery platform. The MTL label was adapted for Arabic reading direction and displayed per-serving nutrients and % recommended daily intake. Treatment effects were estimated using ordinary least squares regressions with robust standard errors and covariate adjustment. **Results:** Of 395 randomized participants, 360 were included in primary analyses (control *n* = 183; MTL *n* = 177). MTL exposure was not associated with a significant change in the primary outcome (basket weighted average MTL score per serving; β = 0.037; *p* = 0.64) or in per-serving calories and nutrients of concern (all *p* > 0.17). In the post-shop assessment, only 47.2% of participants correctly interpreted MTL labels, indicating modest objective label comprehension under the study conditions. **Conclusions:** These findings suggest that the impact of front-of-pack labels likely depends on both implementation features and consumer understanding, and that pairing labels with public communication and nutrition literacy initiatives may be necessary to maximize the effectiveness of labels in Bahrain and the wider Gulf region.

## 1. Introduction

The global burden of non-communicable diseases (NCDs) continues to rise, posing an increasing strain on public health and health system sustainability [1]. Poor diet quality is a leading modifiable risk factor for obesity and NCDs [2]. The Global Burden of Disease Study 2017 estimated that dietary risks were associated with 11 million deaths and 255 million disability-adjusted life years (DALYs) globally [2,3].

The Kingdom of Bahrain is not immune to these global trends. National data show that 72.4% of adults are overweight or obese, 14.7% live with diabetes, and 33.6% have hypertension [4,5,6]. Cardiovascular disease alone accounts for 32% of all deaths in the Kingdom [7]. These outcomes are linked to dietary patterns characterized by low fruit and vegetable intake and high consumption of red and processed meats, sugar, and salt [8]. In response, Bahrain and other Gulf Cooperation Council (GCC) countries are considering population-level nutrition policies to improve dietary habits and reduce the burden of diet-related chronic diseases.

Front-of-pack (FOP) nutrition labeling is one such strategy, widely adopted in high- and middle-income countries to provide simplified, interpretive nutrition information at the point of purchase. These labels can take various forms, including positive labels (e.g., Singapore’s Healthier Choice Symbols, Swedish Keyhole), nutrient-specific warning labels (e.g., Chile’s black stop signs), and graded summary labels (e.g., France’s Nutri-Score) [9,10,11,12]. Among these, the Multiple Traffic Light (MTL) system, originally developed and implemented in the United Kingdom, has received particular attention in the Gulf Cooperation Council (GCC) countries, including Bahrain.

MTL labels assign a color code—green, amber, or red—to indicate low, medium, or high levels of four key nutrients (sugar, fat, saturated fat, and sodium) per 100 g or 100 mL. The label aims to provide consumers with intuitive, nutrient-specific guidance while avoiding the strongly negative framing seen in warning labels. Some countries, including Saudi Arabia and the United Arab Emirates, have adopted the MTL label as part of their broader public health nutrition strategy [13,14,15]. However, experimental evidence on the effectiveness of MTL labels specifically in Arab and Gulf populations remains limited [16,17].

**Figure 1:**
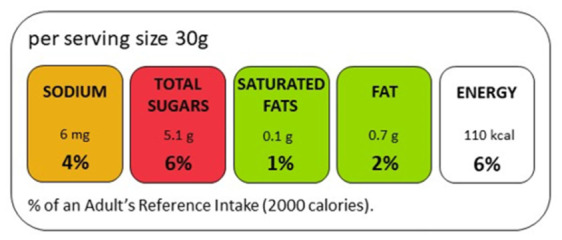
An example of Multiple Traffic Light labels used for the study.

The objective of this study was to test whether an adapted MTL label improves the nutritional quality of grocery purchases among Bahraini consumers. Rather than testing the label as used in other countries, we modified it to better suit the Bahraini context and improve consumer comprehension. The nutrient information was presented from right to left, aligning with Arabic reading direction, and displayed per serving alongside the percentage of recommended daily intake (Figure 1). This adapted label design—including the Arabic reading direction and per-serving presentation—was developed in consultation with the Bahrain Ministry of Health for the purpose of this study. The per-serving format, rather than per 100 g/mL, was adopted to facilitate direct comparisons between products with different recommended serving sizes.

Despite growing evidence on FOP label effectiveness in Western contexts, evidence from Arab-majority and Gulf populations remains limited. Given that label effectiveness is context-specific, shaped by cultural norms, dietary patterns, and consumer literacy, findings from Europe or Latin America may not translate directly to the Gulf region. This study addresses that gap by providing experimental evidence on MTL label effectiveness among Bahraini consumers. Using this modified label, we conducted a two-arm randomized controlled trial via an intercept survey that asked consumers to shop as they normally do, but using a modified online grocery store designed for research purposes. The study is designed to inform national discussions on MTL adoption and contribute to the growing regional evidence base on FOP policies. The remainder of the paper is structured as follows: Section 2 describes the study design, platform, intervention, and analytical approach; Section 3 presents the results; and Section 4 discusses the findings and their implications for FOP labeling policy in Bahrain and the wider Gulf region.

## 2. Materials and Methods

### 2.1. Experimental Design

#### Online Grocery Store

This study was conducted using a Bahrain e-Mart, a web store developed specifically for behavioral nutrition research. It was designed to closely resemble a typical commercial grocery website, while offering full experimental control to test the effects of dietary interventions on consumer behavior.

Participants could freely browse, add, or remove products from their digital shopping cart, and view the total cost of their selections. Standard sorting functions were available, with the default display organized from lowest to highest price. The platform included 3134 food and beverage products commonly sold in Bahraini supermarkets, each accompanied by product images, local-language names, retail prices, descriptions, and nutrition facts (accessible via clickable links).

**Figure 2:**
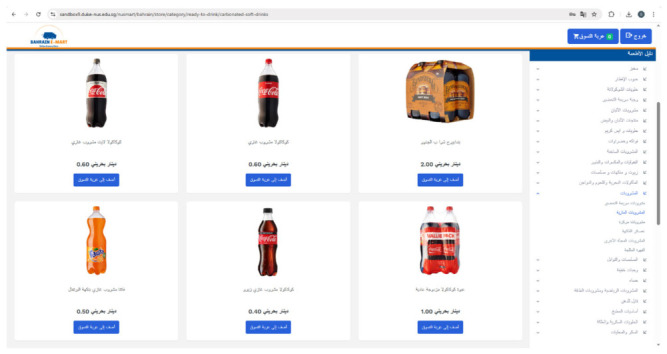
A screenshot of the Arabic version of Bahrain e-Mart (control condition).

Products were grouped into 21 broad food categories and further divided into subcategories (e.g., the “Ready-to-Drink” category included beverage mixes, carbonated soft drinks, and fruit juices, etc.). Figure 2 illustrates the default Bahrain e-Mart interface used in the control condition.

### 2.2. Participants and Procedures

#### 2.2.1. Intervention

The MTL label was adapted for the Bahraini context in two ways: nutrient information was displayed from right to left, aligning with Arabic reading direction, and presented per serving alongside the percentage of the recommended daily intake (%RDI), rather than 100 g/mL. This follows the approach used in the UK MTL label, where color-coding thresholds and displayed nutrient quantities serve distinct purposes: the threshold determines the color signal based on nutrient concentration per 100 g/mL, while the per-serving display communicates actual intake per consumption occasion. Serving sizes were determined from the nutrition facts panels of individual products. To enable direct comparisons within subcategories, a subcategory average serving size was computed and applied uniformly to all products within each subcategory; nutrient content and %RDI displayed on the label were calculated based on this standardized serving size. Traffic light color coding was applied based on per-100 g/mL thresholds, consistent with standard MTL guidelines. RDI values and rounding rules followed the Gulf Cooperation Council Standardization Organization (GSO) Front-of-Pack Traffic Light Nutrition Labeling document, provided by the Bahrain Ministry of Health. This document is based on the UK Department of Health’s Guide to Creating a Front of Pack (FoP) Nutrition Label for Pre-Packed Products Sold Through Retail Outlets (2016) [13]. A detailed example of the calculation of the MTL components is found in the Supplementary Materials.

#### 2.2.2. Recruitment and Procedures

Participants were recruited between January and May 2025 (excluding Ramadan) through intercept surveys conducted at high-footfall public locations such as shopping malls. Trained enumerators invited passersby to participate in a 30 min online grocery shopping study, with the opportunity to win up to 100% cashback on their grocery order. Enumerators approached passersby without any pre-specified selection criteria, and in some cases interested individuals self-approached after seeing study banners displayed at the recruitment booth.

Interested individuals were screened using tablet-based questionnaires in designated seating areas. Eligibility criteria included: (1) age 21 or older (the legal age of adulthood in Bahrain), (2) residency in Bahrain, (3) ability to read and write in Arabic or English, and (4) responsibility for weekly household grocery shopping. Mobile numbers were collected to verify unique participation and process reimbursement. Eligible and consenting participants received a base incentive of Bahrain Diner (BD) 2.5 (≈6.60 USD) upon study completion.

Prior to providing online consent, participants reviewed an information sheet outlining study procedures without disclosing study arms or hypotheses. They were instructed to make purchases for all meals and snacks for their household over the upcoming seven-day period. To encourage participants’ shopping to reflect their usual behaviors, we applied minimum and maximum purchasing thresholds scaled to household size (e.g., BD 10 (≈26.5 USD) per household member, capped at BD 160 (≈424 USD)) and required inclusion of at least four distinct food categories (e.g., dairy, fruits and vegetables). To further anchor choices in real-life behavior, prior to the shopping experiment, participants were told they could receive up to 100% cash back on their selected choices. At checkout, participants spun a digital prize wheel, revealing whether they won the rebate and the size of the rebate (25–100%). Cashback winners were instructed to purchase and submit receipts for the products chosen within 14 days to obtain the rebate. This design incentivized participants to select products they would actually purchase.

After completing online consent and a baseline survey (demographics, household size, education, income, housing type, and self-reported diet-related health conditions), participants were randomized with equal probability into either the MTL or no-label control arm. Randomization sequence was generated by a computer program using a fixed block size of 4, embedded in the grocery platform and concealed from research staff. Following randomization, participants were automatically directed to a randomly selected food subcategory to begin shopping. After completing the shop and spinning the rebate wheel, participants completed a brief post-shop survey. This survey assessed their understanding of the label and its comparability to Nutri-Score and nutrient-specific Warning labels. Enumerators and investigators were blinded to study arm allocation.

The estimated sample size was 450, calculated to detect a standardized effect size of 0.3, with 80% power at a 5% significance level, after accounting for 20% attrition. This effect size, which corresponds to a small to medium effect, is consistent with prior literature of similar evaluations [18,19]. Ethical approval was obtained from the National University of Singapore IRB (Ref: NUS-IRB-2023-852). All procedures adhered to the principles of the Declaration of Helsinki. The trial is registered at ClinicalTrials.gov (ID: NCT06440421, registered 2 June 2024).

### 2.3. Statistical Analyses

#### 2.3.1. Outcome Variables

The primary outcome was the weighted average Multiple Traffic Light (MTL) score per serving of participants’ grocery baskets as a measure of nutritional quality. Higher scores represent greater nutritional quality. Each product was assigned an MTL score based on its levels of four key nutrients—sugar, total fat, saturated fat, and sodium—following the MTL color scheme. Nutrient levels classified as green, amber, and red were scored as 3, 2, and 1, respectively. A product-level average MTL score was calculated, and participant-level scores were derived by weighting each product’s score by the number of servings purchased.

Secondary outcomes included the weighted average per-serving content of calories, sugar, sodium, total fat, and saturated fat across all items in the basket. These measures provided complementary indicators of the overall nutritional quality of the chosen products.

#### 2.3.2. Estimation

We estimated treatment effects using Ordinary Least Squares (OLS) regressions with robust standard errors. The regression specification is as follows:

$$Y_{i}=\beta_{0}+\beta_{1}{MTL}_{i}+X\prime \gamma +\varepsilon_{i}$$

where $Y_{i}$ denotes the outcome for the participant i, the constant term $\beta_{0}$ represents the mean outcome value in the control arm (no-label), and the coefficient $\beta_{1}$ represents the effect of MTL labeling (${MTL}_{i}$). The vector X includes covariates: age, indicators for female, nationality (i.e., Bahraini), high education level (university degree and above), high income (monthly household income of BD 1001 (≈2650 USD) and above), presence of children, household size and body mass index (BMI) as a continuous variable, as well as a dummy for no diet-related health condition (e.g., obesity, diabetes, hypertension) among household members. We also adjusted for participants’ stated prioritization of health as the most important grocery shopping consideration (health consciousness). The main analyses excluded 18 participants whose study completion duration (time from baseline survey initiation to post-study survey completion) was less than the 5th percentile of the distribution (<10 min). Sensitivity analyses re-estimated all models including these participants to assess the robustness of findings.

## 3. Results

### 3.1. Participants

**Figure 3:**
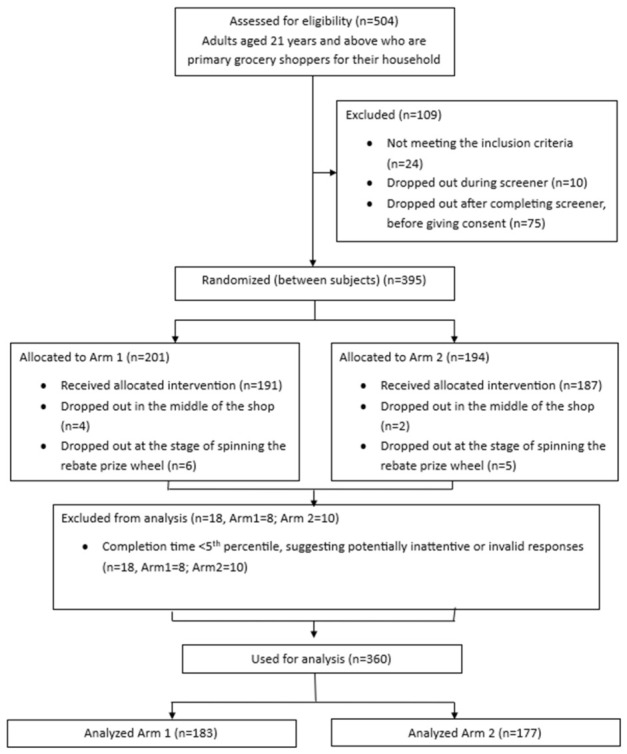
Participant flow diagram.

Figure 3 presents the participant flow through the study. Of 504 individuals who completed the eligibility screener, 395 met the inclusion criteria, provided consent, and were randomized into one of the two study arms. Among these, 379 completed all study components. As described in the Methods, 18 participants with study completion durations below 10 min (the 5th percentile of participation time) were excluded from the primary analyses. The final analytic sample comprised 360 participants.

Table 1 presents the baseline characteristics of participants by study arm and overall. Participants ranged in age from 21 to 77 years, with a mean age of 34.5 years. Slightly fewer than half were female, and approximately 50% held Bahraini citizenship. Educational attainment was generally high in this sample, with 55% reporting a university degree or higher. A total of 39% reported a monthly household income of BD 1001 or more. Nearly half (47%) had children aged 0 to 18 in the household. Thirty-one percent identified health as their primary consideration when grocery shopping, and 42% reported that at least one household member had a diet-related health condition (e.g., diabetes, hypertension, or obesity). Baseline characteristics were statistically balanced across the control and intervention arms.

### 3.2. Effects of MTL Labels on Overall Nutritional Quality and Nutrient Composition of Grocery Purchases

**Figure 4:**
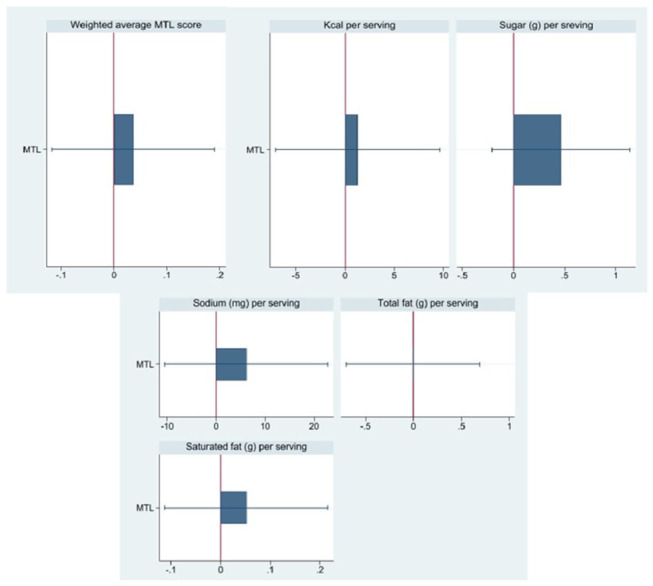
Differences in outcome variables between the Control and MTL arms (95% CI).

Table 2 reports the unadjusted mean and standard deviation of the outcomes in the Control condition, and Figure 4 presents the estimated effects of Multiple Traffic Light (MTL) labels on the nutritional quality and composition of food and beverage purchases. Full regression results are presented in Supplementary Table S1. Exposure to MTL labeling was not associated with statistically significant changes in the primary outcome—weighted average MTL score per serving (β = 0.037, *p* = 0.64). While the coefficient is in the expected direction, the lack of significance suggests that within the current sample, MTL labels were not associated with statistically significant shifts in purchasing behavior toward more healthful nutrient profiles across sugar, fat, saturated fat, and sodium categories.

Similarly, we found no statistically significant differences between the MTL and control arms for secondary nutrient-specific outcomes. The estimated effects were small and imprecise: calories (kcal) per serving (β = 1.291, *p* = 0.76), sugar (g) per serving (β = 0.465, *p* = 0.17), sodium (mg) per serving (β = 6.176, *p* = 0.46), total fat (g) per serving (β = –0.005, *p* = 0.99), and saturated fat (g) per serving (β = 0.052, *p* = 0.53).

Several covariates were significantly associated with healthier purchasing patterns. Older age was linked to a higher average MTL score (β = 0.011, *p* = 0.010) and lower sodium per serving (β = –0.963, *p* = 0.003). Female participants were more likely to purchase products with higher saturated fat content (β = 0.362, *p* < 0.000). Bahraini citizens purchased more sodium per serving than non-citizens (β = 21.696, *p* = 0.027). Households without diet-related health conditions purchased fewer calories (β = −14.049, *p* = 0.002), while health-conscious shoppers purchased less sugar per serving (β = −0.745, *p* = 0.043). In contrast, no statistically significant associations were observed between nutritional quality and higher education, higher income, or having children in the household.

Results for the full sample, including those 18 participants completing the study in less than 10 min, are presented in Supplementary Table S2. Consistent with the main analysis results, we do not find evidence that MTL labels shifted consumers’ food choices towards healthier foods.

### 3.3. Post-Shop Results: Label Perception and Interpretation

As briefly introduced in Section 2, following the shopping task, participants were asked to interpret MTL labels and then to evaluate three front-of-pack (FOP) nutrition labels: Multiple Traffic Light (MTL), Nutri-Score, and Warning Labels.

**Figure 5:**
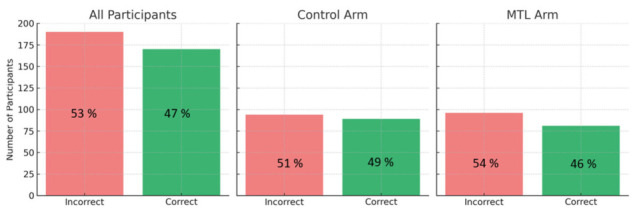
The proportion of participants who correctly paired MTL labels to corresponding food items, by arms and overall.

To assess MTL comprehension, participants were shown various MTL labels and asked to match them with the most appropriate food item (e.g., potato chips, sugar-sweetened beverages). Overall, fewer than half of the participants (47.2%) correctly interpreted the MTL labels, indicating modest levels of objective understanding (Figure 5). Comprehension rates did not statistically differ between the control and MTL arms (χ^2^(1) = 0.30, *p* = 0.585).

**Figure 6:**
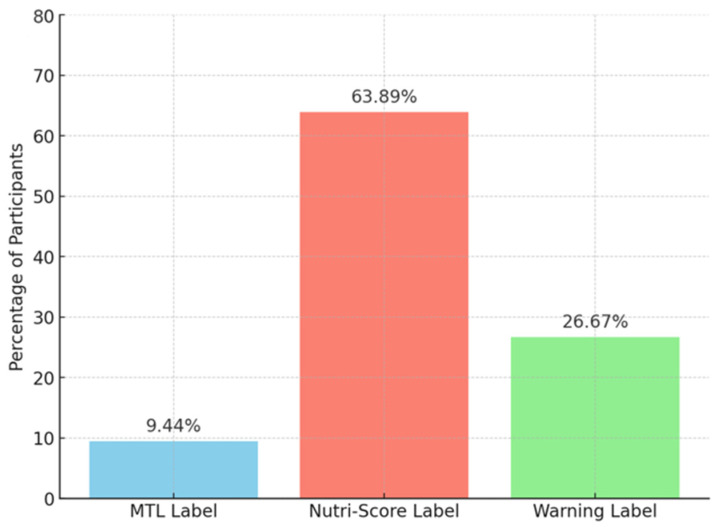
The proportion of participants choosing corresponding labels as the hardest to understand.

When asked which label they found most difficult to understand, a majority of participants (63.9%) identified Nutri-Score, followed by the Warning Label (26.7%). Only 9.4% of participants selected the MTL label as the hardest to interpret (Figure 6).

## 4. Discussion

In this randomized controlled intercept survey trial using an online grocery store in Bahrain, exposure to Multiple Traffic Light (MTL) labels was not associated with a statistically significant improvement in the nutritional quality of grocery purchases, as measured by the weighted average MTL score per serving (β = 0.037, 95% CI [−0.12, 0.19]; *p* = 0.64). MTL labels similarly did not significantly reduce the per-serving content of calories, sugar, sodium, total fat, or saturated fat.

The null effects observed in our study align with a broader body of literature showing that while front-of-pack (FOP) labels can increase the likelihood of selecting healthier products, their impact on calories and select nutrients from purchased products is often small and not clinically meaningful [20]. One plausible contributing factor to the small effects observed in our study is modest objective label comprehension under the study conditions. In the post-study survey, fewer than half of participants (47.2%) correctly interpreted the MTL labels, suggesting that more education may be needed to support effective label use during shopping. This is not unique to Bahrain; evidence from the region suggests that practical nutritional literacy, particularly applying nutrition information to everyday food choices, can be limited in some settings [21], which may attenuate the impact of FOP labels when implemented without accompanying communication. Several covariate associations were also observed—notably, female participants purchased baskets higher in saturated fat per serving—though these are exploratory findings from a convenience sample and should not be over-interpreted in the absence of replication in larger, more representative studies.

MTL labels come with the additional challenge that many products are less healthy in some aspects but healthier in others. Consider an individual who is overweight and suffers from hypertension who is choosing between two products, one higher in calories and the other higher in sodium. The MTL clearly identifies these differences, which may be internalized by consumers, but even a health-conscious consumer would be reasonably confused as to which product to choose. Labels that only show aggregate information, such as Nutri-Score, avoid this confusion but are less effective for consumers looking to improve the consumption of a single nutrient or reduce caloric intake [22]. Warning labels, by contrast, take a different approach: rather than grading nutrient levels across a spectrum, they flag only excess nutrients using a simple binary signal, a design that may be easier for ordinary consumers to interpret [23]. Evidence suggests that warning labels are particularly effective at reducing purchases of products high in a specific nutrient, while Nutri-Score is more effective at improving the overall nutritional quality of purchases [24]—a pattern consistent with findings from our prior study comparing these two label formats among Saudi consumers (Shin et al., 2023 [18]). In this respect, MTL and warning labels may share a similar potential to reduce consumption of specific nutrients of concern, though MTL provides more granular information by assessing each nutrient across three levels—low (green), moderate (amber), and high (red)—rather than issuing a binary warning only for products exceeding a threshold. For this reason, neither labeling approach is clearly superior. Rather, these findings point to consumer education—both on how to interpret labels and on the health implications of specific nutrients—as a promising avenue for future intervention, warranting further investigation.

This study has several limitations. First, although the virtual supermarket was designed to closely mimic real-world shopping, we cannot rule out the possibility of hypothetical shopping biases. While we implemented basket requirements and offered a cashback prize, the extent to which purchases fully reflected participants’ actual shopping behaviors remains uncertain. Second, our study was based on a convenience sample recruited in public venues, with higher income and higher educational attainment than the general population in Bahrain. This suggests that our results may not generalize to other income and education groups and does not allow us to assess differential effects across socioeconomic groups. Third, exposure was limited to a single shopping session. Repeated exposure, combined with nutritional education, may be necessary for labels to influence consumer purchasing patterns and support longer-term habit formation toward healthier products. Fourth, although the virtual environment reduced some uncontrolled variability, it cannot fully capture the dynamics of in-person shopping environments, where time constraints, in-store marketing, product placement, and social factors can further shape choices. In particular, the absence of real-world contextual cues, such as shelf placement, promotional displays, and peer influence, means that the effect of MTL labels observed under controlled platform conditions may not fully generalize to naturalistic shopping settings. Fifth, we did not capture individual-level data on participants’ use of the platform’s sorting functions, specifically, whether participants changed the default lowest-to-highest price ordering during their shopping session. Although the default sorting was identical across both arms and therefore cannot confound the estimated treatment effect, the absence of this data limits our understanding of how price visibility may have shaped product navigation and selection within each arm. More broadly, the prominence of price information in a default lowest-to-highest price ordering display may have made price a more salient decision cue than it would be in a physical retail environment, where products are not arranged by price. This could limit the generalizability of our findings to settings where price salience is lower.

Future studies should evaluate different FOP label formats (e.g., Nutri-Score, warning labels) in larger and more diverse populations and in settings that more closely reflect day-to-day shopping in the Gulf region. In particular, field trials in physical retail environments and longitudinal designs with repeated exposure are needed to assess sustained effects in the presence of strong behavioral drivers (e.g., promotions, product placement, in-store marketing). To identify implementation features that maximize impact, future experiments should systematically vary choice architecture and UX/UI features within online platforms (e.g., label prominence, brief in-context explanations, default sorting mechanisms). Future work should also test the effectiveness of the label on lower-income households and explore label and price interactions, recognizing that the effectiveness of the label may vary depending on product prices and the presence of taxes (subsidies) that discourage (encourage) purchases of healthier (less healthy) products. Furthermore, research should investigate how nutrition and health literacy mediate the effectiveness of FOP labels, and whether combining labeling with select education and marketing programs amplifies their effects. Finally, future studies employing MTL or similar multi-nutrient labeling systems may also consider pre-specifying single-nutrient threshold indicators as supplementary outcomes—for example, defining a product as ‘red’ based on exceeding the threshold for any one nutrient—provided such definitions are established prior to data collection to avoid post hoc definitional ambiguity.

## 5. Conclusions

In conclusion, MTL labeling did not materially change the nutritional quality of grocery purchases in this study, likely due to modest consumer literacy in interpreting the label. These findings suggest that the impact of front-of-pack labels depends on both implementation features and consumer understanding. Whether pairing labels with public communication and nutrition literacy initiatives would maximize effectiveness in Bahrain and the wider Gulf region remains an important hypothesis for future research.

## Figures and Tables

**Table 1:** Summary statistics of the participants by arms and total (*N* = 360).

	Total	1 (Control)	2 (MTL)	Difference (1–2) *p*-Value
	*N* = 360	*N* = 183	*N* = 177	
Age (years), mean (SD)	34.50 (11.43)	34.27 (10.74)	34.74 (12.12)	0.70
Female, *n* (%)	142 (39.4%)	75 (41.0%)	67 (37.9%)	0.54
Bahraini, *n* (%)	187 (51.9%)	94 (51.4%)	93 (52.5%)	0.82
Household size, mean (SD)	4.2 (2.0)	4.1 (2.0)	4.2 (1.9)	0.37
Having Children, *n* (%)	170 (47.2%)	88 (48.1%)	82 (46.3%)	0.74
High educational level (university degree and above), *n* (%)	197 (54.7%)	100 (54.6%)	97 (54.8%)	0.98
High household income (monthly income BD 1001 and above), *n* (%)	140 (38.9%)	69 (37.7%)	71 (40.1%)	0.64
Health-conscious, *n* (%)	113 (31.4%)	57 (31.1%)	56 (31.6%)	0.92
Household with no underlying health condition, *n* (%)	209 (58.1%)	108 (59.0%)	101 (57.1%)	0.71
BMI, mean (SD)	26.1 (4.8)	25.7 (4.7)	26.5 (4.9)	0.11

Note: continuous variables were compared between arms using independent samples *t*-tests, and categorical variables using Pearson chi-square tests.

**Table 2:** Unadjusted mean and standard deviation of the primary and secondary outcomes in the Control condition.

Measure	Mean	Std. Dev.
Weighted average of MTL score per serving	9.67	(0.74)
Average kcal per serving in a basket	126.43	(41.16)
Average sugar per serving in a basket	4.62	(3.32)
Average sodium per serving in a basket	83.68	(81.06)
Average total fat per serving in a basket	5.36	(3.34)
Average saturated fat per serving in a basket	1.24	(0.79)

## Data Availability

The datasets used and/or analyzed during the current study are not publicly available due to a lack of consent from all participants for making the data publicly available, but they are available from the corresponding author upon reasonable request.

## Supplementary Materials

The following supporting information can be downloaded at: https://www.mdpi.com/article/10.3390/nu18101645/s1, Table S1. Effects of MTL labels on Nutritional Quality and Nutrient Composition of Grocery Purchases; Table S2. Effects of MTL labels on Nutritional Quality and Nutrient Composition of Grocery Purchases (Full sample). Supplementary Note S1: Example Calculation of MTL Label Values. Supplementary Note S2: Normality test.

## Abbreviations

The following abbreviations are used in this manuscript:

NCDs	Non-communicable diseases
DALYs	disability-adjusted life years (DALYs)
FOP	Front-of-pack
BD	Bahrain Diner
MTL	Multiple-traffic light
BMI	Body Mass Index
