# Supplementary material for: A Randomized Intercept Survey Trial to Test the Effectiveness of Multiple Traffic Light Labels on Online Grocery Shopping Behaviors in Bahrain
Source: Nutrients. 2026 May 21;18(10):1645. doi: 10.3390/nu18101645 (PMC13209343; doi:10.3390/nu18101645)

Table S1. Effects of MTL labels on Nutritional Quality and Nutrient Composition of Grocery Purchases.

	(1) weighted average of MTL score per serving	(2) average kcal per serving in a basket	(3) average sugar per serving in a basket	(4) average sodium per serving in a basket	(5) average total fat per serving in a basket	(6) average saturated fat per serving in a basket
MTL	0.037	1.291	0.465	6.176	-0.005	0.052
	(0.08)	(4.23)	(0.34)	(8.47)	(0.35)	(0.08)
Age	0.011***	0.335*	-0.028*	-0.963***	-0.006	-0.001
	(0.00)	(0.20)	(0.02)	(0.32)	(0.02)	(0.00)
Female	-0.093	8.637*	-0.123	17.259*	0.159	0.362***
	(0.08)	(4.62)	(0.39)	(8.83)	(0.37)	(0.09)
Bahraini	-0.092	5.375	-0.518	21.696**	-0.652	0.179
	(0.10)	(5.05)	(0.44)	(9.79)	(0.41)	(0.11)
HH size	-0.013	-1.316	0.166	1.023	-0.050	-0.021
	(0.03)	(1.30)	(0.11)	(2.99)	(0.11)	(0.03)
Having Kids	-0.059	1.253	0.375	-0.781	0.032	0.032
	(0.09)	(4.81)	(0.43)	(9.70)	(0.39)	(0.11)
High Educ	0.037	2.903	0.164	-2.526	-0.181	0.086
	(0.08)	(4.45)	(0.36)	(8.52)	(0.37)	(0.08)
High income	0.028	-2.004	-0.128	-10.920	0.236	0.138
	(0.08)	(4.60)	(0.35)	(9.29)	(0.37)	(0.09)
No health condition	-0.104	-14.384***	0.429	-2.514	-0.121	-0.082
	(0.09)	(4.65)	(0.38)	(9.94)	(0.38)	(0.09)
Health-Conscious	0.060	1.944	-0.745**	2.247	-0.074	-0.001
	(0.09)	(4.86)	(0.37)	(7.77)	(0.40)	(0.09)
BMI	-0.012	-0.001	-0.045	0.311	0.044	0.008
	(0.01)	(0.48)	(0.04)	(1.04)	(0.04)	(0.01)
Constant	9.777***	119.876***	5.930***	90.184**	4.969***	0.815***
	(0.28)	(16.21)	(1.25)	(34.88)	(1.26)	(0.28)
R-squared	0.054	0.063	0.061	0.058	0.014	0.097
Observations	360	360	360	360	360	360

Notes: Robust standard errors are reported in parentheses. Statistical significance denoted by * p<0.1. ** p<0.05, *** p<0.01.

Table S2. Effects of MTL labels on Nutritional Quality and Nutrient Composition of Grocery Purchases (Full sample).

	(1) weighted average of MTL score per serving	(2) average kcal per serving in a basket	(3) average sugar per serving in a basket	(4) average sodium per serving in a basket	(5) average totalfat per serving in a basket	(6) average saturatedfat per serving in a basket
MTL	0.052	2.545	0.352	6.747	-0.021	0.027
	(0.08)	(4.23)	(0.33)	(8.23)	(0.34)	(0.08)
Age	0.010**	0.264	-0.030*	-0.955***	-0.006	-0.003
	(0.00)	(0.20)	(0.02)	(0.32)	(0.02)	(0.00)
Female	-0.093	7.823*	-0.157	16.525*	0.134	0.335***
	(0.08)	(4.57)	(0.37)	(8.69)	(0.36)	(0.09)
Bahraini	-0.083	8.890*	-0.532	24.780**	-0.592	0.236**
	(0.09)	(5.11)	(0.43)	(9.89)	(0.40)	(0.11)
HH size	-0.016	-1.687	0.180	-0.174	-0.039	-0.031
	(0.02)	(1.30)	(0.11)	(2.99)	(0.10)	(0.03)
Having Kids	-0.068	1.081	0.319	-0.743	0.097	0.018
	(0.09)	(4.77)	(0.42)	(9.44)	(0.38)	(0.10)
High Educ	0.039	4.902	0.195	-1.151	-0.138	0.090
	(0.08)	(4.43)	(0.35)	(8.32)	(0.36)	(0.08)
High income	0.041	-3.065	-0.013	-8.119	0.150	0.129
	(0.08)	(4.61)	(0.35)	(9.16)	(0.37)	(0.09)
No health condition	-0.090	-13.360***	0.479	-0.106	-0.211	-0.063
	(0.08)	(4.62)	(0.37)	(9.82)	(0.37)	(0.09)
Health-Conscious	0.064	2.648	-0.597*	1.291	-0.087	0.016
	(0.08)	(4.75)	(0.36)	(7.44)	(0.38)	(0.09)
BMI	-0.013	0.130	-0.031	0.188	0.050	0.009
	(0.01)	(0.48)	(0.04)	(0.99)	(0.04)	(0.01)
Constant	9.808***	116.961***	5.554***	94.922***	4.756***	0.896***
	(0.27)	(16.09)	(1.25)	(33.31)	(1.23)	(0.27)
R-squared	0.055	0.061	0.054	0.056	0.013	0.095
Observations	378	378	378	378	378	378

Notes: Robust standard errors are reported in parentheses. Statistical significance denoted by * p<0.1. ** p<0.05, *** p<0.01.

Supplementary Note S1: Example Calculation of MTL Label Values

The following example illustrates how serving sizes, nutrient values, traffic light colour codes, and %RDI were computed for two products in the Carbonated Soft Drinks subcategory (Ready-to-Drink category): 7-Up (1,980 ml pack) and Coca-Cola Regular Twin Pack (3,000 ml pack).

Step 1 — Serving size standardisation

Serving sizes were first taken from individual product nutrition facts panels: 355 ml for 7-Up and 100 ml for Coca-Cola Regular Twin Pack. A subcategory average serving size was then computed across all products in the Carbonated Soft Drinks subcategory and found to be 276 ml. This standardised serving size was applied uniformly to both products, enabling direct nutritional comparisons regardless of manufacturer-declared serving sizes.

Step 2 — Nutrient values per 100 ml

Nutrient content per 100 ml was calculated from each product's nutrition facts panel and rounded following GSO FDS 2233 rounding rules. For 7-Up: energy = 39 kcal (rounded to nearest 1 kcal); total sugars = 9.9g (< 10g, rounded to nearest 0.1g); total fat = 0g (≤ 0.5g, declared as 0g); saturated fat = 0g (≤ 0.1g, declared as 0g); sodium = 0.01g (< 1g and > 0.005g, rounded to nearest 0.01g). For Coca-Cola Regular Twin Pack: energy = 42 kcal; total sugars = 11g (≥ 10g, rounded to nearest 1g); total fat = 0g; saturated fat = 0g; sodium < 0.005g (declared as 0g).

Step 3 — Traffic light colour assignment

Colour thresholds were applied based on nutrient content per 100 ml, following GSO FDS 2233 beverage standards. For sodium, the displayed sodium value (mg) was first converted to salt equivalents (g) using the formula: salt (g) = sodium (mg) × 2.5 ÷ 1,000, and the salt threshold was applied to assign the colour. The displayed value on the MTL label, however, was sodium in mg per serving, consistent with the nutrition facts panel format.

Nutrient	Threshold (per 100 ml)	7-Up	Coca-Cola
Total sugars	Green ≤2.5g / Amber >2.5g to ≤11.25g / Red >11.25g	Amber (9.9g)	Amber (11g)
Total fat	Green ≤1.5g / Amber >1.5g to ≤8.75g / Red >8.75g	Green (0g)	Green (0g)
Saturated fat	Green ≤0.75g / Amber >0.75g to ≤2.5g / Red >2.5g	Green (0g)	Green (0g)
Salt	Green ≤0.3g / Amber >0.3g to ≤0.75g / Red >0.75g	Green (0.027g)	Green (0.005g)

Step 4 — Displayed nutrient values and %RDI per standardised serving (276 ml)

Nutrient amounts per 276 ml serving were calculated and rounded following GSO FDS 2233 rounding rules. %RDI was computed using GSO reference values: energy 2,000 kcal; total fat 70g; saturated fat 20g; total sugars 90g; salt 6g (sodium RDI derived as 2,400 mg). Displayed values are as follows:

Nutrient	7-Up per 276 ml	%RDI	Coca-Cola per 276 ml	%RDI
Energy (kcal)	107	5%	116	6%
Total sugars (g)	27	30%	29	32%
Total fat (g)	0	0%	0	0%
Saturated fat (g)	0	0%	0	0%
Sodium (mg)	30	1%	6	0%

Note: although traffic light colour coding for sodium used salt equivalents (per Step 3), the value displayed on the label is sodium in mg per serving. Both products receive the same traffic light colour profile: amber for total sugars, green for total fat, saturated fat, and sodium.

Supplementary Note S2: Normality test

We have tested the normality of residuals using the Shapiro-Wilk test, which yielded W = 0.992 (p = 0.047). Although this is a marginal rejection at the 5% level, the W statistic of 0.992 is exceptionally close to 1, indicating that the actual shape of the residuals is likely very close to normal. With n = 360, the Shapiro-Wilk test has sufficient power to reject the null even when departures from normality are negligibly small and inconsequential for inference. The Q-Q plot confirms this interpretation: the residuals lie almost perfectly along the 45-degree reference line across the entire distribution, with no meaningful deviation at the tails. The residuals are therefore normally distributed for all practical purposes.

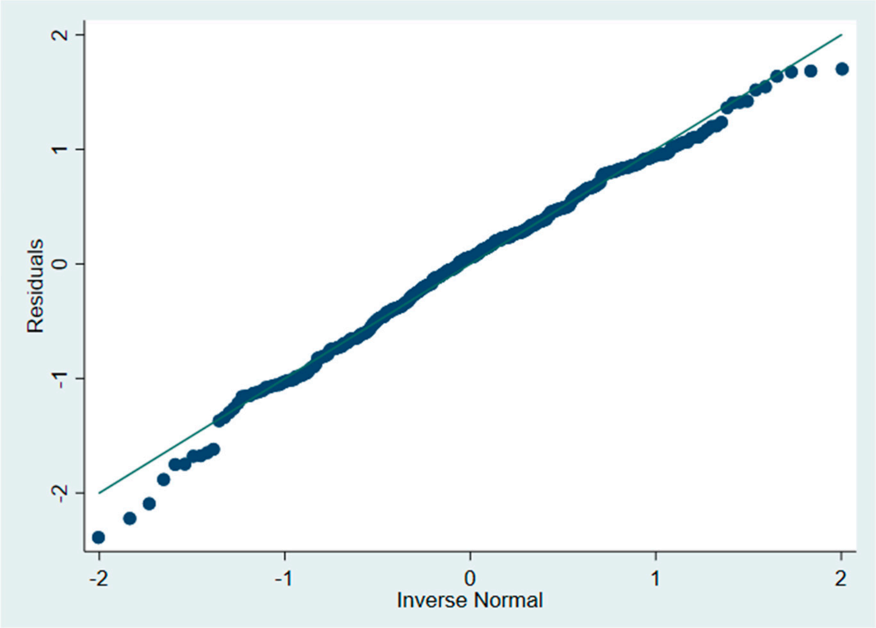

Supplement: Supplementary file 1 [file nutrients-18-01645-s001.zip › nutrients-4188489-supplementary.pdf]
